# Isolation of Batborne Neglected Zoonotic Agent Issyk-Kul Virus, Italy

**DOI:** 10.3201/eid3004.231186

**Published:** 2024-04

**Authors:** Davide Lelli, Ana Moreno, Sabrina Canziani, Laura Soliani, Maya Carrera, Anna Castelli, Francesca Faccin, Tiziana Trogu, Enrica Sozzi, Gian Luca Cavallari, Matteo Mauri, Fabiana Ferrari, Cristian Salogni, Chiara Garbarino, Chiara Chiapponi, Marco Farioli, Antonio Lavazza

**Affiliations:** University of Parma via Gramsci, Parma, Italy (D. Lelli);; Istituto Zooprofilattico Sperimentale della Lombardia e dell’Emilia Romagna, Brescia, Italy (D. Lelli, A. Moreno, S. Canziani, L. Soliani, M. Carrera, A. Castelli, F. Faccin, T. Trogu, E. Sozzi, C. Salogni, C. Garbarino, C. Chiapponi, A. Lavazza);; Wildlife Rehabilitation Center WWF of Valpredina, Cenate Sopra, Italy (G.L. Cavallari, M. Mauri);; Piacenza Wildlife Rescue Centre, Piacenza, italy (F. Ferrari);; DG Welfare Regione Lombardia, Milano, Italy (M. Farioli)

**Keywords:** Issyk-kul virus, bats, zoonosis, Hypsugo savii, Orthonairovirus, viruses, vector-borne infections, Italy

## Abstract

We isolated Issyk-Kul virus (ISKV) from a bat sampled from Italy in 2021 and conducted ISKV-specific surveillance in bats collected in Italy during 2017–2023. ISKV circulation among synanthropic and sedentary species of bat, such as Savi’s pipistrelle bat (*Hypsugo savii*) in northern Italy, may have public health implications in this region.

Issyk-Kul virus (ISKV), family *Nairoviridae*, was first isolated in 1970 from a noctule bat (*Nyctalus noctule*) trapped near Lake Issyk-Kul, Kyrgyzstan (*1*). ISKV was subsequently detected in bats of several countries in central Asia and in *Ixodes vespertilionis* and *Argas vespertilionis* ticks (*2*). ISKV has caused sporadic outbreaks of illness in humans, characterized by fever, headaches, myalgia, and nausea (*2*,*3*). Bats and ticks are assumed to be reservoirs of ISKV; transmission to humans is associated with tick bites and exposure to bat feces and urine (*2*,*4*,*5*). Moreover, *Aedes caspius* mosquitoes, common in Europe and central Asia, may have a role as vectors, having been considered competent through experimental infection (*6*,*7*).

Portions of ISKV genome were detected in northern bats (*Eptesicus nilssonii*) in Germany (*4*) and a Brandt’s bat (*Myotis brandtii*) in Sweden (*8*), suggesting that the ISKV geographic range expanded to Europe. We isolated and performed whole-genome characterization of ISKV detected in a Savi’s pipistrelle bat (*Hipsugo savii*) (hereafter referred to as Savi’s bat) in Italy in 2021 and present the results of ISKV-specific surveillance of 415 bats collected during 2017–2023.

Ethics review and approval were waived for this study, which did not involve animal killing or suffering. Samples were collected exclusively from animals that died in wild recovery centers in the context of the regional surveillance plans for wildlife. Therefore, we believe that it does not fall in the provisions of the national law (e.g., DLSG 4/3 2014, n. 26—Application at national level of the EU Directive 2010/63/UE), and no ethics approval or permit for animal experimentation was required.

## The Study

We isolated the virus from an adult female Savi’s bat that spontaneously died in a wildlife recovery center in northern Italy. The bat was originally found alive on August 17, 2021, in Bergamo Province, northern Italy, by a private citizen who brought it to the center. Clinically, the bat exhibited lethargy, inappetence, and weight loss. It died 11 days after admission to the center, and no trauma or macroscopic pathologic lesions indicative of infectious disease were observed at necropsy. DNA barcoding confirmed the species as Savi’s bat. We collected organ samples (e.g., lung, heart, liver, spleen, intestine, and brain) for laboratory investigations focused mainly on virus detection.

**Figure 1:**
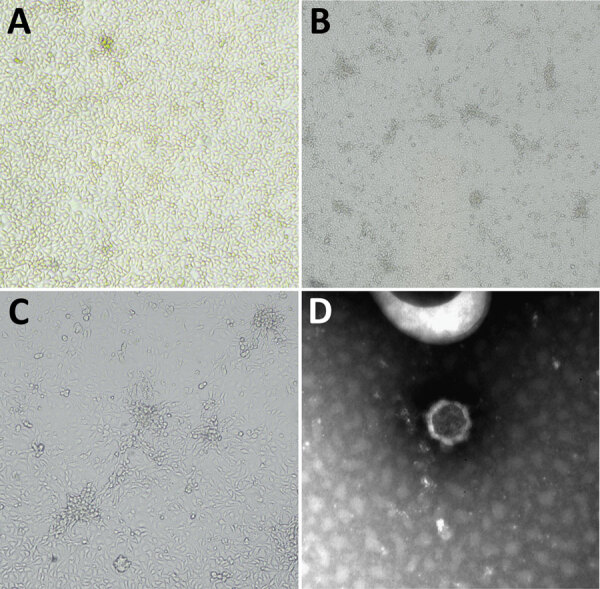
Microscopic appearance of Issyk-Kul virus IT-297348-34/2022, isolated from a *Hypsugo savii* bat, in study of batborne neglected zoonotic agent Issyk-Kul virus, Italy. A) Issyk-Kul–infected MARC 145 cells, mock infection; original magnification ×10. B) Issyk-Kul–infected MARC 145 cells showing cytopathic effect at 120 hours after infection; original magnification ×4. C) Issyk-Kul–infected MARC 145 cells showing cytopathic effect at 120 hours after infection; original magnification ×10. D) Negative-staining electron microscopy performed on cell supernatants (NaPT 2%), showing a viral particle of 55–60 nm morphologically referable to nairovirus; original magnification ≈x550,000

We assessed the brains of all bats for negativity to rabies and related lyssaviruses by using real-time PCR (*9*). We isolated a virus on MARC 145 cells (fetal monkey kidney) inoculated with a pool of viscera (lung, heart, liver, spleen). Cytopathic effect was noted 5 days after inoculation during the secondary passage and was characterized by cell monolayer degeneration with isolated foci of rounded and aggregated cells (Figure 1, panels A–C). Furthermore, electron microscopy performed on cell culture supernatants revealed distinct viral particles of 55–60 nm, morphologically referable to a nairovirus (Figure 1, panel D).

The complete genome sequence of isolated virus IT-297348–34/2022 (IT-ISKV), obtained through a standardized next-generation sequencing protocol (*10*), revealed the 3 typical nairovirus genome segments: large (L) (11,978 nt), medium (M) (4,907 nt), and small (S) (1,457 nt). The highest nucleotide identity for each gene segment was to ISKV strains detected in a Brandt’s bat (*Myotis brandtii*) and bat-associated ticks in Sweden (strain Sun-2020, k99_1658, k99_589), and in northern bats (*Eptesicus nilssonii*) in Germany (strain PbGER) (Table 1). We submitted the complete genome sequences to GenBank (accession nos. OR583909–11).

**Table 1:** Highest nucleotide sequence identities for each protein of IT-ISKV isolated in study of batborne neglected zoonotic agent Issyk-Kul virus, Italy*

Gene	% Similarity (query cover, %)	ISKV strain	Host	Country (year)	GenBank accession no.	Reference
Large	95.44 (99)	K_k99_1658_len_12288	Bat-associated tick	Sweden (2020)	OP514654	Unpub. data
	95.34 (99)	LEZ 86–787	*Carios* *vespertilionis* tick	Germany (1986)	KR537441	(*11*)
	95.31 (99)	Sun-2020	*Myotis brandtii* bat	Sweden (2020)	OP380632	(*8*)
Medium	81.55 (72)	Sun-2020	*M. brandtii* bat	Sweden (2020)	OP380631	(*8*)
	81.11 (78)	k99_589	Bat-associated tick	Sweden (2020)	OP804626	Unpub. data
	81.34 (71)	LEIV-315K	*Nyctalus noctula* bat	Kyrgyzstan (1973)	KR709220	(*3*)
Small	97.51 (90)	PbGER	*Eptesicus nilssonii* bat	Germany (2008–2011)	MW275296	(*4*)
	89.11 (100)	Sun-2020	*M. brandtii* bat	Sweden (2020)	OP380630	(*8*)
	89.04 (100)	LEZ 86–787	*C. vespertilionis* tick	Germany (1986)	KR537443	(*11*)

*IT-ISKV, Issyk-Kul virus IT-297348-34/2022.

The phylogenetic tree, based on the complete L genome sequences of viruses in the genus *Orthonairovirus,* assigned IT-ISKV to the Keterah genogroup. That genogroup includes the few available sequences of ISKVs detected in bats and ticks in Sweden, Germany, and central Asia, as well as other sequences of Keterah virus (detected in bats from Malaysia), Uzun Agach virus (in bats from Kazakhstan), and Gossas virus (in bats from Senegal) (Figure 1) (*11*). Phylogenetic trees constructed with complete S and M genes showed similar results with the same topology because of the small number of ISKV sequences available in the genome databases (Appendix Figures 1, 2). 

**Figure 2:**
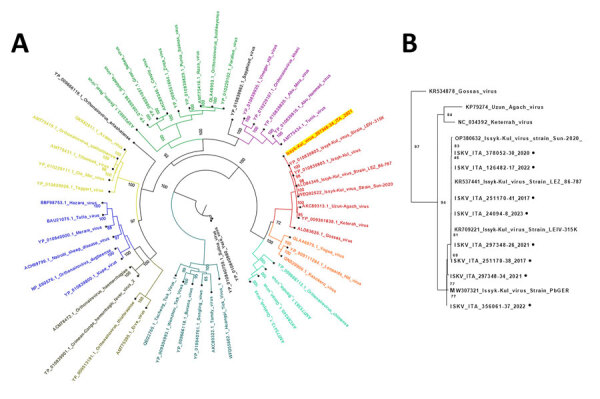
Phylogenetic analysis of isolates from study of batborne neglected zoonotic agent Issyk-Kul virus, Italy A) Phylogeneny nairovirus protein sequences for *Orthonairovirus* large (L) segments, including the complete sequence obtained from *Hypsugo savii* bats, highlighted in yellow. Sequence colors were based on the genogroups proposed by Ozeki et al. 2022 (*12*). B) Nucleotide alignment magnification of a short PCR-targeted region of the L segment, encompassing all sequences derived from bat surveillance conducted during 2017–2023 identified as Issyk-Kul virus IT-297348-34/2022. Numbers along branches indicate bootstrap values.

After detecting IT-ISKV in the Mediterranean area, we developed and standardized ISKV-specific endpoint reverse transcription PCR targeting the L gene (Appendix) to enhance knowledge of the ISKV ecology and screen its diffusion in bat populations. We used ISKV-specific endpoint reverse transcription PCR to detect viral RNA in the necropsied tissues and to screen cultured cells. We performed Sanger sequencing of generated amplicons to confirm ISKV RNA.

During 2017–2023, we collected 415 bats representing 13 species in the Lombardy and Emilia-Romagna regions of northern Italy. The bats originated mainly from wildlife recovery centers, which usually receive rescued bats (usually near human settlements), or bats found dead during passive surveillance. Most of the bats examined were Savi’s bats and Kuhl’s bats (*Pipistrellus kuhlii*) (Appendix Table 1). We tested sampled organs (lung, heart, liver, spleen, intestine) for ISKV. We detected 8 bats positive by PCR for ISKV; 7 were Savi’s bats and 1 was a whiskered bat (*Myotis mystacinus*) (Table 2), and they were recovered in 2017, 2020, 2021, 2022, and 2023 (Appendix Table 2). We constructed a phylogenetic tree based on the partial L genome sequences with all ISKVs detected in Italy (Figure 2, panel B). None of the ISKV-positive bats had ticks attached, and the ticks (*Ixodes vespertilionis*) found on the analyzed bats were ISKV negative.

**Table 2:** Issyk-Kul–positive bats and associated GenBank accession number assigned to the gene sequences detected in study of batborne neglected zoonotic agent Issyk-Kul virus, Italy*

Sample	Year	Bat species	Origin	Virus isolation (cell culture)	Sequence	GenBank accession no.	Nucleotide similarity (%)
251170-38/2017	2017	*Hypsugo savii*	WRC WWF Valpredina. Bergamo, Italy	No	Partial L gene	OR583901	Issyk-Kul virus LEZ 86–787 (98.79)
251170-41/2017	2017	*H. savii*	WRC WWF Valpredina. Bergamo, Italy	No	Partial L gene	OR583902	Issyk-Kul virus LEZ 86–787 (99.27)
378052-30/2020	2020	*H. savii*	WRC WWF Valpredina. Bergamo, Italy	No	Partial L gene	OR583903	Issyk-Kul virus K_k99_1658_len_12288 (99.17)
297348-34/2022	2021	*H. savii*	WRC WWF Valpredina. Bergamo, Italy	Yes	Full genome	OR583909–11, OR583905	Issyk-Kul virus/Prackenbach bat nairovirus (96.65)
297348-26/2022	2021	*H. savii*	WRC WWF Valpredina. Bergamo, Italy	No	Partial L gene	OR583904	Issyk-Kul virus LEZ 86–787 (99.42)
126482-17/2022	2022	*H. savii*	WRC Piacenza, Iataly	No	Partial L gene	OR583906	Issyk-Kul virus LEZ 86–787 (99)
356061-37/2022	2022	*H. savii*	WRC WWF Valpredina. Bergamo, Italy	No	Partial L gene	OR583907	Issyk-Kul virus/Prackenbach bat nairovirus (98.42)
24094-8/2023	2023	*Myotis mystacinus*	WRC Piacenza, Italy	No	Partial L gene	OR583908	Issyk-Kul virus LEZ 86-787 (98.91)

*L, large; WRC, wildlife recovery center; WWF, World Wildlife Fund.

## Conclusions

Tickborne orthonairoviruses may be agents of human emerging infectious diseases (*13*). Crimean-Congo hemorrhagic fever virus is the most notable pathogen in the genus *Orthonairovirus* because of its public health effect with high fatality rates and widespread geographic distribution (*14*). However, several other emerging and neglected orthonairoviruses, such as ISKV, can cause clinical nonlethal diseases in humans (*15*).

Our isolation and characterization of IT-ISKV showed high L and S gene identity to the ISKV strains detected in Sweden, Germany, and central Asia (*3*,*4*,*8*). However, the level of M gene nucleotide similarity to the other known ISKV strains (80.98%–81.55%) suggests that IT-ISKV could represent a new ISKV strain from the Mediterranean area, most likely derived from an assortment with a yet unknown virus.

In that context, we conducted ISKV-specific surveillance among bats collected during 2017–2023 with the aim of determining the presence and diffusion of the virus in bat populations in northern Italy. Findings suggest that ISKV in that area seem to be associated with Savi’s bats and whiskered bats*,* which may represent a previously unrecognized source for ISKV transmission to other wildlife species, ticks, and humans, as has already happened elsewhere (*2*,*3*). Savi’s bats are a synanthropic and sedentary species that roost in buildings and represent the most common bat species in urban areas, suggesting possible public health implications.

ISKVs identified in bats in Italy were detected from a pool of organs, as in the previously described ISKV PbGER (*4*), which was found predominantly in the liver, spleen, and lung tissues, indicating systemic infection of bats instead of mere passaging of intestinal tick content. Future investigations may provide information about virus tissue distribution and pathogenesis by using histopathology and may define the infection prevalence in the bat populations through serologic tests.

The successful cell-culture isolation of IT-ISKV suggests the possible shedding of infectious virus particles, which represents a crucial point for assessing viral zoonotic risks that may emerge from synanthropic bats. Such results indicate the emergence of this neglected zoonotic agent in the Mediterranean area, which might have public health relevance because of its potential transmission to humans. Raising awareness of the risks deriving from this zoonosis should suggest adoption of specific surveillance and prevention programs for ISKV and other nairoviruses at the human–wild animal interface.

Appendix Additional information for study of batborne neglected zoonotic agent Issyk-Kul virus, Italy.
