# Supplementary material for: Isolation of Batborne Neglected Zoonotic Agent Issyk-Kul Virus, Italy
Source: Emerg Infect Dis. 2024 Apr;30(4):786–90. doi: 10.3201/eid3004.231186 (PMC10977857; doi:10.3201/eid3004.231186)
Supplement: Appendix — Additional information for study of batborne neglected zoonotic agent Issyk-Kul virus, Italy. [file 23-1186-Techapp-s1.pdf]

Article DOI: https://doi.org/10.3201/eid3004.2311186

EID cannot ensure accessibility for supplementary materials supplied by authors. Readers who have difficulty accessing supplementary content should contact the authors for assistance.

Isolation of Bat-Borne Neglected Zoonotic Agent Issyk-Kul Virus, Italy

Appendix

Materials and Methods

Sampling

From January 2017 to January 2023, 415 bats were analyzed in the context of surveillance implemented in Northern Italy regarding emerging viruses of bats, which was launched in 2010 (*1*,*2*). Dead bats of different species were collected for virological investigations from wild animal rescue/rehabilitation centers. The bat species were taxonomically identified by both morphologic characteristics according to the European bat identification keys (*3*) and by a molecular method (*4*). The anamnestic data were gathered when available. The bat carcasses were necropsied, the tissue samples were collected, and subjected to a diagnostic protocol broadly targeting viral agents.

Virus isolation and Electron Microscopy

After necropsy, organ samples (lungs, heart, kidney, brain, and intestines) were homogenized in minimal essential medium (1 g/10 mL), which contained antibiotics, and were centrifuged at 3000 g for 15 min. Samples were inoculated in confluent monolayers of MARC 145 and VERO (African green monkey) cells, incubated at 37°C with 5% CO2, and observed daily for 7 days for cytopathic effects (CPEs). In the absence of CPEs, the cryo-lysates were subcultured twice onto fresh monolayers. The supernatant from cell cultures showing CPEs was submitted for viral identification with negative-staining electron microscopy (nsEM) by using the Airfuge method (*5*) (Airfuge, Beckman Coulter, Inc. Life Sciences, Indianapolis, Indiana, USA). The examination was done using a Tecnai G2 Spirit Biotwin transmission electron microscope (TEM; FEI, Hillsboro, Oregon, OR, USA) operating at 85 kV. The observation was

done at 13,500–43,000x for at least 15 min before being considered negative. Identification and recognition of the observed viral particles were based on their morphology.

End-point one-step RT-PCR for ISKV detection

An endpoint one-step RT-PCR was developed using the complete viral genome sequenced. Primers IZSLER-ISKV F (5′- CTAGCTCTGCTGATTATGAG −3′) and IZSLER-ISKV R (5′- GCCAAGATAGTTGCGTCAATC −3′) were designed on a conserved 844 bp region of Keterah Genogroup L gene, which encodes for the RNA-dependent RNA polymerase of the ISKV. Total RNA was extracted from samples using QIAsymphony DSP Virus/Pathogen Mini Kit, following the manufacturer's instructions. The PCR assay was performed with the QIAGEN® OneStep RT-PCRmaster mix (Qiagen, Hilden, Germany). The reaction mixture contained 0.6 pmol of sense and antisense primer, Qiagen buffer 1X, 400 μM of each dNTPs, Qiagen OneStep RT-PCR Enzyme mix 1μl, 8U of RNase inhibitor and 5 μl of the RNA template in a final volume of 25 μl. PCR conditions were optimized using homogenized bat organs and cell culture samples, which were known to be positive and negative for ISKV infections. The thermal cycling conditions consisted of 30 min at 50°C for reverse transcription, 15 min at 95°C for the initial enzyme activation, and 40 thermal cycles of 94°C for 60 s; 59°C for 60 s; and 72°C for 30 s, with a final elongation step of 72°C for 10 min. Amplicons were visualized on 2% agarose electrophoretic gels stained with EuroSafe Fluorescent Nucleic Acid Stain (Euroclone, Milan, Italy) used at a 1× concentration.

Positive samples were confirmed by Sanger sequencing using the IZSLER ISKV forward and reverse primers.

Phylogenetic Analysis

A collection of nairovirus complete protein sequences was downloaded based on the classification provided by the International Committee on Taxonomy of Viruses (ICTV), including all defined member species of the genus Orthonairovirus, available as of July 1, 2023. The sequences were aligned using the CLUSTAL algorithm (*6*). For the L segment, a nucleotide alignment of a targeted region (positions 368–760) was performed using the same algorithm. A maximum-likelihood tree was generated using Iqtree-1.6.12 (*7*), with the best-fit model automatically selected by ModelFinder. The statistical significance of the tree topology was

assessed using ultrafast bootstrap with 1000 replicates. Tree visualizations were generated using FigTree v.1.1.4.

References

1. Lelli D, Moreno A, Lavazza A, Bresaola M, Canelli E, Boniotti MB, et al. Identification of Mammalian orthoreovirus type 3 in Italian bats. Zoonoses Public Health. 2013;60:84–92. PubMed https://doi.org/10.1111/zph.12001

2. Lelli D, Papetti A, Sabelli C, Rosti E, Moreno A, Boniotti MB. Detection of coronaviruses in bats of various species in Italy. Viruses. 2013;5:2679–89. PubMed https://doi.org/10.3390/v5112679

3. Dietz C, von Helversen O. Illustrated identification key to the bats of Europe. Electronic publication. Version 1.0. Released 15.12.2004. Tuebingen & Erlangen (Germany).

4. De Benedictis P, Leopardi S, Markotter W, Velasco-Villa A. The Importance of Accurate Host Species Identification in the Framework of Rabies Surveillance, Control and Elimination. Viruses. 2022;14:492. PubMed https://doi.org/10.3390/v14030492

5. Lavazza A, Pascucci S, Gelmetti D. Rod-shaped virus-like particles in intestinal contents of three avian species. Vet Rec. 1990;126:581. PubMed

6. Sievers F, Wilm A, Dineen D, Gibson TJ, Karplus K, Li W, et al. Fast, scalable generation of high-quality protein multiple sequence alignments using Clustal Omega. Mol Syst Biol. 2011;7:539. PubMed https://doi.org/10.1038/msb.2011.75

7. Nguyen LT, Schmidt HA, von Haeseler A, Minh BQ. IQ-TREE: a fast and effective stochastic algorithm for estimating maximum-likelihood phylogenies. Mol Biol Evol. 2015;32:268–74. PubMed https://doi.org/10.1093/molbev/msu300

8. Ozeki T, Abe H, Ushijima Y, Nze-Nkogue C, Akomo-Okoue EF, Ella GWE, et al. Identification of novel orthonairoviruses from rodents and shrews in Gabon, Central Africa. J Gen Virol. 2022;103. PubMed https://doi.org/10.1099/jgv.0.001796

Appendix Table 1. List of bat species analyzed in the study

Species	N°	%
Eptesicus serotinus	1	0.2
Hypsugo savii	170	41
Myotis emarginatus	1	0.2
Myotis crypticus	1	0.2
Myotis daubentonii	9	2.2
Myotis mystacinus	4	1.0
Nyctalus leisleri	3	0.7
Pipistrellus kuhlii	151	36.4
Pipistrellus nathusii	6	1.5
Pipistrellus pipistrellus	15	3.6
Plecotus uratus	3	0.7
Plecotus macrobullaris	1	0.2
Tadarida teniotis	4	1.0
Plecotus sp.	2	0.5
Vespertilio murinus	1	0.2
Pipistrellus spp.	43	10.4
Total	415	100

Appendix Table 2. Number of bats analyzed in the study divided by year of sampling

Year	N° tested bats	N° positive bats
2017	98	2
2018	42	0
2019	30	0
2020	117	1
2021	58	2
2022	52	1
2023	18	2
Total	415	8

Appendix Table 3. Details of the ISKV-positive bats in the study: clinical signs, organs tested and positive

N° Identification	Year	Species	Age	Sex	Clinical signs	Sampled organs	Positive samples
251170/38	2017	*Hypsugo savi*	J	n.a.	No signs	I + V	I + V
251170/41	2017	*Hypsugo savi*	J	n.a.	No signs	I + V	I + V
378052/30	2020	*Hypsugo savi*	SA	F	No signs	I + V	I + V
297348/26	2021	*Hypsugo savi*	SA	M	Dehydration	I + V	I + V
297348/34	2021	*Hypsugo savi*	SA	F	Apathy, inappetence weight loss	I + V	I + V
126482/17	2022	*Hypsugo savi*	J	F	No signs	I + V	V
356061/37	2023	*Hypsugo savi*	n.a.	n.a.	No signs	I + V	I
24094/8	2023	*Myotis mystacinus*	A	M	Traumatic lesions (cat aggression)	I + V	I + V

A = adult, SA = sub-adult (this-year-born individuals, usually ranging in age from 1 to 10–11 mo) J = juvenile, - recently born, incapable of flight, M = male, *F* = female, I = intestine, V = pool of viscera (heart + lung + spleen + liver)
n.a. = not available.

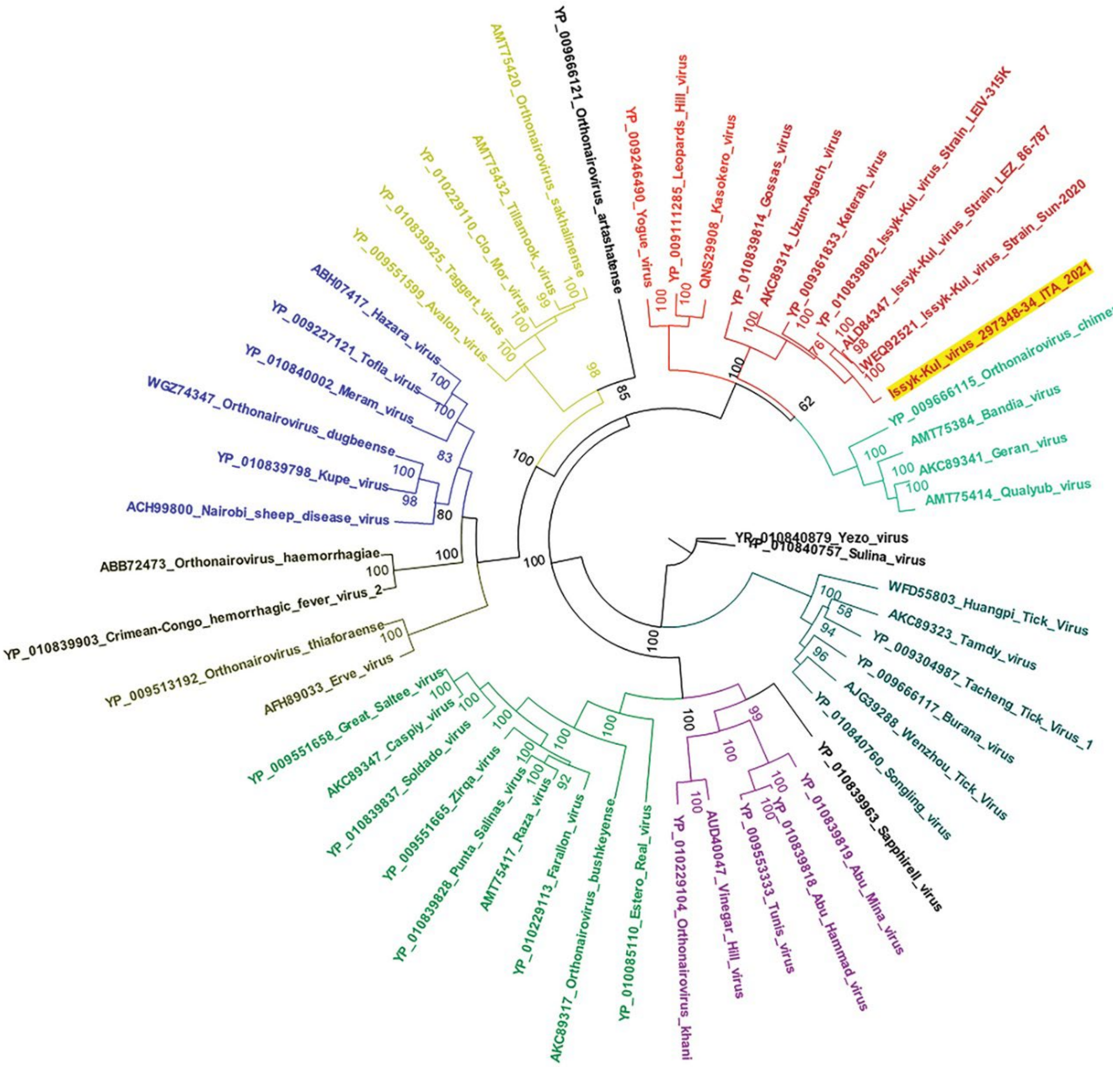

Appendix Figure 1. Phylogenetic analysis of nairovirus protein sequences for the M segments, including our complete sequence obtained from *Hypsugo savii*, highlighted. The sequences were colored based on the genogroups proposed by Ozeki *et al*. 2022 (*8*).

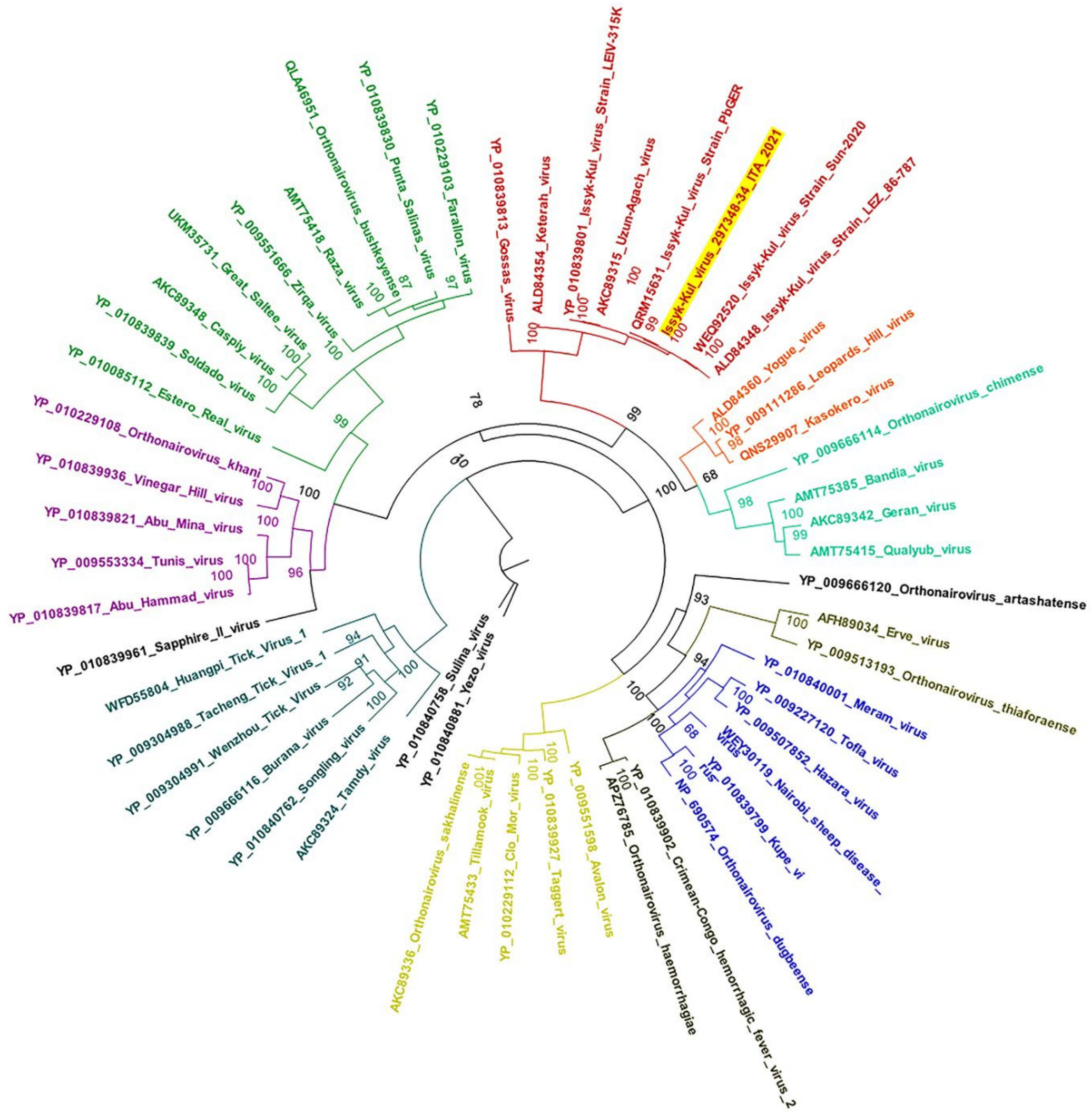

Appendix Figure 2. Phylogenetic analysis of nairovirus protein sequences for S segments, including our complete sequence obtained from *Hypsugo savii*, highlighted. The sequences were colored based on the genogroups proposed by Ozeki *et al*. 2022 (*8*).
